# Exploring the Impact of Semaglutide on Cognitive Function and Anxiety-Related Behaviors in a Murine Model of Alzheimer’s Disease

**DOI:** 10.3390/biomedicines12122689

**Published:** 2024-11-25

**Authors:** Ianis Kevyn Stefan Boboc, Petrica-Daniel Dumitrelea, Andreea Daniela Meca, Liliana Mititelu-Tartau, Maria Bogdan

**Affiliations:** 1 Department of Pharmacology, Faculty of Pharmacy, University of Medicine and Pharmacy of Craiova, 200349 Craiova, Romania; kevynboboc@gmail.com (I.K.S.B.); andreea_mdc@yahoo.com (A.D.M.); 2 Doctoral School, University of Medicine and Pharmacy of Craiova, 200349 Craiova, Romania; 3 Department of Pharmacology, Faculty of Medicine, “Grigore T. Popa” University of Medicine and Pharmacy, 700115 Iasi, Romania

**Keywords:** semaglutide, Alzheimer’s disease, murine models, open field test, novel object recognition test, social chamber test, 0-maze test

## Abstract

Background: Alzheimer’s disease (AD), the most prevalent form of dementia, is characterized by progressive cognitive decline and behavioral disturbances, with an increasing incidence as the global population ages. This study investigates the effects of semaglutide (SEM), a glucagon-like peptide-1 analog, on cognitive function and anxiety-like behavior in a transgenic murine model of AD. Methods: 20 mice were randomly distributed into the following groups (*n* = 5): (WT + VEH) group: C57BL/6J + saline, (WT + SEM) group: C57BL/6J + semaglutide, (AD + VEH) group: AD + saline, (AD + SEM) group: AD + semaglutide. The animals underwent a four-week treatment, during which we monitored blood glucose levels, body weight, and responses in an open field test, novel object recognition test, social chamber test, and 0-maze test. Results: Post-treatment, SEM significantly reduced blood glucose levels in AD mice, aligning them with those of wild-type controls. Cognitive assessments indicated an improvement in the investigation index for SEM-treated mice compared to those receiving a vehicle, suggesting cognitive benefits. Although SEM did not significantly enhance motor and exploratory activities, it displayed a potential anxiolytic effect, particularly evident in the combined anxiety index, with notable differences observed before and after treatment in the AD group. Conclusions: The findings of this pilot study suggest that SEM may play a dual role in managing AD by improving glycemic control and potentially enhancing cognitive function. As the landscape of AD treatment evolves, the comprehensive approach of utilizing SEM could pave the way for innovative interventions targeting the complex interplay of metabolic and cognitive dysfunctions in this challenging neurodegenerative disorder.

## 1. Introduction

Alzheimer’s disease (AD) is the most common form of dementia worldwide, responsible for up to 70% of dementia cases, and is a chronic, progressively debilitating neurodegenerative disorder. AD manifests through memory loss, cognitive decline, and a gradual impairment in daily functional abilities [1,2]. Pathologically, AD is characterized by the presence of amyloid-β (Aβ) plaques and tau protein neurofibrillary tangles in the brain, which disrupt neural communication and lead to widespread neuronal death [3,4]. It is estimated that AD will affect 152 million people by 2050 [5], and the WHO has recognized it as a global public health priority [5,6]. AD is divided into two primary forms: familial AD (early-onset), a rare form affecting approximately 2% of cases and often resulting from specific genetic mutations, and sporadic AD (late-onset), the more common form which typically appears later in life and lacks a clear genetic cause [7,8]. Although our understanding of AD has advanced significantly, its underlying pathogenesis remains incompletely understood, and there are currently no treatments that halt or reverse disease progression, underscoring the urgent need for new therapeutic approaches [9].

Semaglutide (SEM) is a glucagon-like peptide-1 (GLP-1) analog with a structural homology of 94% to human GLP-1 [10]. Originally developed for the management of type 2 diabetes mellitus (DM), SEM has demonstrated substantial efficacy in controlling blood glucose levels and promoting weight loss. It is available as an injectable formulation and an oral tablet [11]. The FDA granted approval for SEM’s injectable form in 2017 and its oral form in 2019 for type 2 DM management. In 2021, SEM also received FDA approval for chronic weight control, with corresponding EMA approvals in 2018 (injectable) and 2020 (oral) [12,13].

The dosing regimen for SEM varies between its formulations. The injectable form is administered once weekly, beginning with an initial dose of 0.25 mg for the first four weeks, which is then increased to 0.5 mg and subsequently to a maintenance dose of 1.0 mg per week. The oral formulation of SEM is taken daily, starting at 3 mg per day for the first 30 days, and then increasing to 7 mg, with a maximum dose of 14 mg per day [13]. Both forms have demonstrated similar profiles of efficacy and safety, offering flexibility based on patient preference and clinical need [12].

Beyond its primary metabolic benefits, SEM has demonstrated anti-inflammatory properties, contributing to reductions in systemic inflammation [14,15,16]. These effects are beneficial not only for DM management but also in the context of other chronic conditions such as cardiovascular diseases, neurodegenerative disorders, and inflammatory bowel disease. Since inflammation is now recognized as a crucial factor in both the development and exacerbation of various chronic conditions, including AD, there is increasing interest in exploring SEM’s anti-inflammatory properties in neurodegenerative diseases [17]. This interest is further supported by emerging research that links AD with DM, suggesting that medications like SEM may also hold potential benefits for cognitive function and AD symptom management [18].

To better understand AD pathogenesis and develop new therapeutic strategies, experimental models of AD, particularly transgenic mouse models, are extensively utilized. These models can replicate a variety of AD-related pathologies, including the formation of Aβ plaques and other neurodegenerative markers [19,20]. Using these models, researchers can study disease progression and test potential therapies in a controlled environment, providing critical insights into mechanisms that could translate into human applications.

In this study, we sought to evaluate the effects of SEM on multiple behavioral and physiological parameters in a murine model of AD. Specifically, we assessed SEM’s impact on learning and memory, social interactions, and anxiety-like behaviors, as these areas are significantly affected in AD. Additionally, we monitored blood glucose levels and body weight, as these metabolic factors are central to SEM’s pharmacological effects and may indirectly influence cognitive and behavioral outcomes in AD models. Through this research, we aimed to explore SEM’s therapeutic potential in AD, building on existing evidence of its metabolic advantages.

This study contributes to the growing body of evidence that supports the potential benefits of metabolic interventions in the treatment of AD. By investigating the combined effects of SEM on metabolic management, cognitive function, and behavioral health, our research provides a preliminary but critical insight into the multi-targeted therapeutic approach that could pave the way for more effective treatment strategies for AD. The study’s findings will serve as a pilot investigation for future, larger-scale studies that can more conclusively assess the efficacy of this approach. Furthermore, the research methodology employed in this study can serve as a model for subsequent investigations aiming to explore the combined impact of pharmacological and behavioral interventions in the treatment of neurodegenerative diseases.

## 2. Materials and Methods

The experiments were conducted at the Experimental Research Center for Normal and Pathological Aging from the University of Medicine and Pharmacy in Craiova, Romania. All national and European norms regarding the ethics and handling of laboratory animals were strictly followed. The procedures to which the laboratory animals were subjected received the approval of the Ethics Committee of the University of Medicine and Pharmacy in Craiova (No. 266/29.11.2023).

### 2.1. Animals

This study included 20 mice, randomly distributed into the following groups (*n* = 5):

- Group (WT + VEH): C57BL/6J + saline;
- Group (WT + SEM): C57BL/6J + semaglutide;
- Group (AD + VEH): AD + saline;
- Group (AD + SEM): AD + semaglutide.

Of these mice, 10 belonged to the C57BL/6J strain (wild type, WT), while the other 10 mice were transgenic, presenting AD pathology, with a positive test for amyloid precursor protein (APP positive) and presenilin (PS) mutations [21]. The animals were provided by the Biobase of the University of Medicine and Pharmacy in Craiova, and presented an average weight of 29 g, 6 months of age with ad libitum access to food and water, with a day/night cycle of 12 h/12 h from 07:00 to 19:00, and an ambient temperature of 21 °C and air humidity 60%. Three days before the start of the experiment, mice were removed from the quarantine area to acclimatize within the new environment.

The 20 mice were randomly assigned to the four experimental groups using a simple randomization method. Ten WT mice were numbered consecutively from 1 to 10. Then, a random number generator was used to assign each mouse to one of the two groups: WT + VEH, WT + SEM. The same procedure was applied to the 10 transgenic mice for the two groups: AD +VEH, AD +SEM. Randomization was conducted prior to the start of the experiment to eliminate any bias in group allocation.

Before starting the injection procedure, the mice were weighed to calculate the requirement of the active substance, using a high-precision electronic scale (Scale, CJ-600, HBI Technologies, Phoenix, AZ, USA) capable of allowing the animals to move freely within a semi-open space.

**Figure 1:**
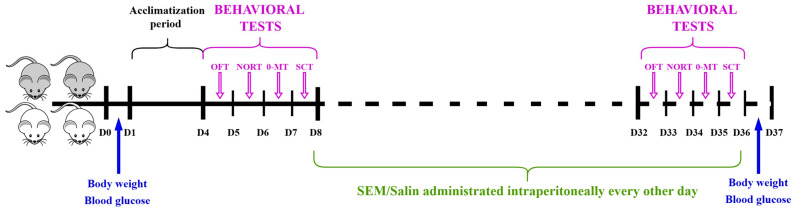
Time schedule for experimental design.

SEM solution prepared extemporaneously by diluting the substance with sterile saline was used. Mice from the treatment groups received 0.1 mg/kg SEM solution, while the control group received sterile saline (0.9% NaCl solution) [22]. SEM solution was injected intraperitoneally, every other day, for a period of 28 days, at the same hour (Figure 1) [22].

### 2.2. Blood Glucose Monitoring

Blood glucose levels were measured both before starting the treatment and upon its completion. Mice were gently secured in a manually crafted polypropylene tube, with their tails exposed. A small prick was made at the tip of each tail using a sterile needle, and gentle “milking” from the base to the tip of the tail produced a drop of blood [23]. This blood drop was then applied directly to the glucometer strip (Contour Plus^®^, Ascensia Diabetes Care, Basel, Switzerland) to obtain glucose readings.

### 2.3. Weight Monitoring

Weight monitoring was conducted prior to and at the end of the treatment using a high-precision electronic scale (Scale, CJ-600, HBI Technologies), allowing the animals free movement within a semi-open space.

### 2.4. Behavioral Studies

Behavioral tests, except for the 0-MT, were recorded and analyzed using an automated tracking system (EthoVision XT 17, Noldus Technology, Wageningen, The Netherlands). Animals were acclimated to the testing room 30 min prior to the experiment. At the end of each test, the arena was cleaned with 75% ethanol to remove residual odors. Testing was conducted both before and after the pharmacological treatment (Figure 1) [24].

#### 2.4.1. Open Field Test (OFT)

The OFT is a common measure of exploratory behavior and general activity in rodents [25,26]. The OFT was conducted as outlined in previous studies. To summarize, the test consisted of positioning the mice in the center of a rectangular arena measuring 15 cm in height, 55 cm in length, and 30 cm in width, where they were given the opportunity to explore for a duration of 10 min. Throughout this exploration phase, both locomotor activity and anxiety-related behaviors were observed and assessed. Key parameters recorded during the test included the total distance traveled, movement speed, and the amount of time spent in the arena’s central area [24,25,26].

#### 2.4.2. Novel Object Recognition Test (NORT)

NORT is frequently used to assess the cognitive function of mice, investigating various aspects of learning and memory in mice [27,28,29]. Initially, the animals were placed in an arena measuring 15 cm in height, 55 cm in length, and 30 cm in width, where two identical objects were positioned at equal distances of 15 cm from the side walls for a duration of 6 min. After this initial phase, the animals were returned to their cages for 1 h. Following this period, the animals were reintroduced into the same arena, but one of the identical objects was substituted with a novel object. The animals were then permitted to explore this new configuration for another 6 min. The percentage of total investigation time was calculated using the formula, which served as an indicator of short-term memory performance [24,27,28,29]:

$$\%=\frac{Tn}{Tn+Tf\;}\;\times 100$$

%—percentage of total investigation time

*Tn*—time spent with the new object

*Tf*—time spent with the familiar object

#### 2.4.3. Social Chamber Test (SCT)

The SCT evaluates social deficits and social recognition in rodents by utilizing their natural social behaviors to assess their social preferences [30,31]. The testing arena comprised three equal-sized chambers (15 cm high, 40 cm long, and 20 cm wide) that were interconnected, allowing for the quantification of both social approach and recognition. Each chamber features a square opening at the bottom, enabling mice to move freely between the compartments. In the side chambers, two wire cages were positioned, leaving the center chamber empty [31].

During the habituation phase, mice were initially confined to the center chamber for 10 min. Following this, they were given another 10 min to explore all three chambers to familiarize themselves with the environment. For the sociability assessment, the test mouse was placed in the center chamber, with one side of the chamber housing a stainless-steel wire cage containing a same-age mouse, while the other side chamber held an empty wire cage. The test mouse was then allowed to explore the entire setup for 10 min [31].

During the social novelty phase, a new, unfamiliar mouse was introduced into the previously empty cage, while the familiar mouse remained in its original cage. The test mouse was then allowed an additional 10 min to explore and interact with both the familiar and novel mice [30,31,32].

To calculate the sociability index, a straightforward method involves comparing the time the test animal spent with the unfamiliar mouse to the time spent in the empty compartment. The sociability index is determined using the following formula:

$$\mathrm{SI}=\frac{Tm1-Tc}{Tm1+Tc}$$

SI = sociability index

*Tm_1_* = time spent in the chamber with the mouse 1

*Tc* = time spent in the chamber with the empty cage

A positive sociability index indicates a preference for social interaction, a zero index suggests no preference for social interaction or non-specific exploration, and a negative index signifies an avoidance of social interaction.

For the social novelty index, which compares interactions between the two mice, the formula is:

$$\mathrm{SC}=\frac{Tm1-Tm2}{Tm1+Tm2}$$

SC = social novelty

*Tm_1_* = time spent in the chamber with the mouse 1

*Tm_2_* = time spent in the chamber with the mouse 2

A positive index indicates that the test animal prefers interacting with mouse 1 over mouse 2, a zero index denotes no preference between the two mice, and a negative index suggests a preference for interacting with mouse 2 over mouse 1.

#### 2.4.4. 0-Maze Test (0-MT)

The 0-MT is an unconditioned anxiety test. It was developed as a modification of the plus maze test and has been validated to assess anxiety in mice [33].

The apparatus consists of a circular maze divided into four equal quadrants, alternating between open arms (sections without walls) and closed arms (sections with walls). The maze has the following dimensions: a standing height of 61 cm, a wall height of 20 cm, a diameter of 50 cm, and a track width of 5 cm. The animal is placed in the center of this enclosed space and allowed to explore for 5 min. The amount of time spent in the open sections is considered the primary scoring parameter, as increased time in these areas is interpreted as a reduction in anxiety [34]. Additionally, the number of transitions between the open and closed arms is recorded.

The anxiety index (IA) was calculated according to the following formula:

$$\mathrm{IA}=\frac{Toa}{Ttm}\;\times 100$$

IA = anxiety index

*Toa* = time spent in open arms

*Ttm* = total time in maze

Low IA values (close to 0) indicate avoidant behavior and high anxiety while high IA values (close to 100) indicate exploratory behavior and low anxiety. The Combined Anxiety Index (IAC) was created to provide a more sensitive tool for detecting potential deficiencies in animals. This index considers both the time spent in the open arms and the number of transitions between open and closed arms, calculated as follows:

$$\mathrm{IAC}=\frac{\left(\frac{Toa}{Ttm})+(\frac{Nt}{Nmax}\right)}{2}\;\times 100$$

IAC = Combined Anxiety Index

*Toa* = time spent in open arms

*Ttm* = total time in maze

*Nt* = number of transitions between open and closed arms

*Nmax* = maximum number of transitions observed across all subjects

Low IAC values (close to 0) indicate high anxiety and avoidance while high IAC values (close to 100) indicate low anxiety and greater exploration.

### 2.5. Statistical Data Processing

The obtained data were expressed as the arithmetic mean ± standard deviation (SD) of the mean values and statistically analyzed using GraphPad Prism 10 (GraphPad Software, San Diego, CA, USA) and Microsoft Excel 2024. Differences in means among the groups were analyzed with two-way repetitive ANOVA (Sidak’s Multiple comparation test) with Geisser–Greenhouse correction, after the data set passed normality test (Shapiro–Wilk test and Kolmogorov–Smirnov test). Statistical significance was defined as *p*-values less than 0.05 (* *p* < 0.05, ** *p* < 0.01, *** *p* < 0.001 and **** *p* < 0.0001).

## 3. Results

### 3.1. Blood Glucose Monitoring

**Figure 2:**
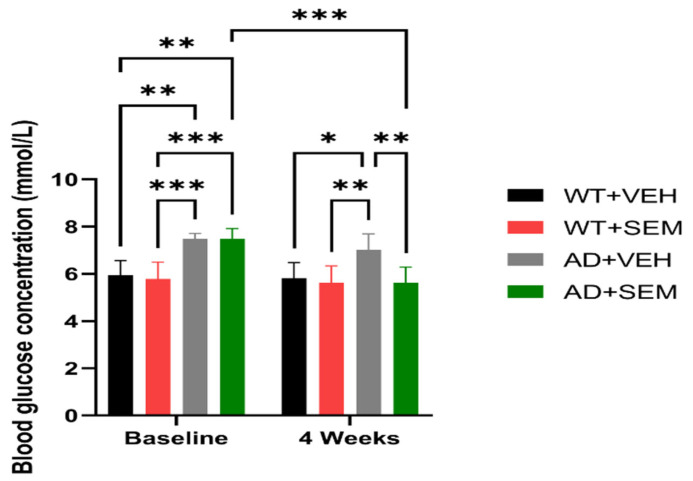
Monitoring of blood glucose levels in mice before and after SEM/vehicle therapy. * *p* < 0.05, ** *p* < 0.01, *** *p* < 0.001, data are presented as Mean ± SD.

The animals in the WT groups show different glycemic values compared to the positive APP animals since baseline: WT + VEH (5.94 ± 0.62 mmol/L) vs. AD + VEH (7.48 ± 0.22 mmol/L), *p* = 0.0020; WT + VEH (5.94 ± 0.62 mmol/L) vs. AD + SEM (7.49 ± 0.42 mmol/L), *p* = 0.0019; WT + SEM (5.79 ± 0.70 mmol/L) vs. AD + VEH (7.48 ± 0.22 mmol/L), *p* = 0.0006; WT + SEM (5.79 ± 0.70 mmol/L) vs. AD + SEM (7.49 ± 0.42 mmol/L), *p* = 0.0006 (as seen in Figure 2).

After 4 weeks, the glycemic levels of APP-positive animals treated with SEM (5.62 ± 0.66 mmol/L) aligned closely with those of the WT + VEH group (5.82 ± 0.65 mmol/L) and the WT + SEM group (5.63 ± 0.70 mmol/L). Notably, significant differences were found between the APP-positive animals treated with the vehicle (7.01 ± 0.68 mmol/L) compared to the WT + VEH group (5.82 ± 0.65 mmol/L, *p* = 0.0231) and the WT + SEM group (5.63 ± 0.70 mmol/L, *p* = 0.0060). Additionally, APP-positive animals receiving SEM exhibited a statistically significant reduction in blood glucose levels (5.62 ± 0.66 mmol/L, *p* = 0.0060) compared to those that received only the vehicle (7.01 ± 0.68 mmol/L). Furthermore, there was a statistically significant difference between the baseline measurements and the values at the 4-week mark for APP-positive animals treated with SEM, with initial blood glucose levels at the start of treatment being 7.49 ± 0.42 mmol/L and decreasing to 5.62 ± 0.66 mmol/L by the end of the 4 weeks (*p* = 0.0005).

### 3.2. Weight Monitoring

No statistical differences were noted among the four groups of animals prior to the commencement of SEM treatment; however, APP-positive animals tended to exhibit lower weights compared to the WT animals.

**Figure 3:**
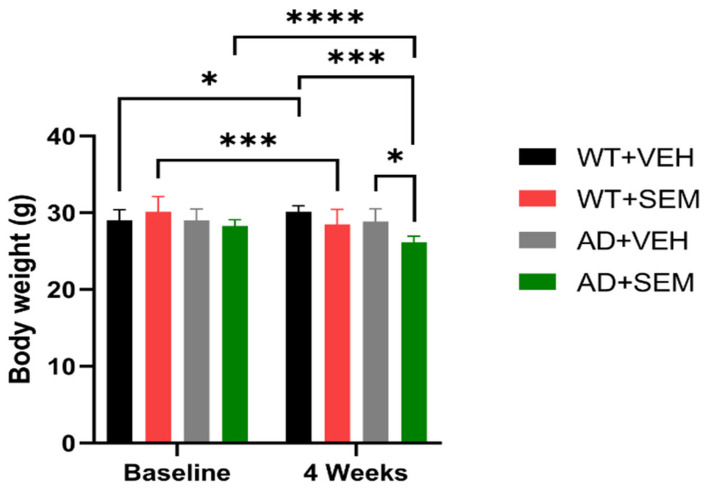
Monitoring of animals weight before and after SEM/vehicle therapy. * *p* < 0.05, *** *p* < 0.001, **** *p* < 0.0001, data are presented as Mean ± SD.

Significant weight gains were recorded in the WT + VEH group from baseline (29.02 ± 1.39 g) to the 4-week mark (30.12 ± 0.80 g), with a *p*-value of 0.0183. In contrast, weight reductions were observed in both the WT + SEM group (30.10 ± 2 g at baseline vs. 28.47 ± 1.96 g at 4 weeks, *p* = 0.0007) and the AD + SEM group (28.27 ± 0.81 g at baseline vs. 26.15 ± 0.78 g at 4 weeks, *p* < 0.0001). After 4 weeks, there were statistically significant differences in weight loss between the AD + SEM group (26.15 ± 0.78 g) and the AD + VEH group (28.86 ± 1.66 g), with a *p*-value of 0.0348, as well as between the AD + SEM group and the WT + VEH group (30.12 ± 0.80 g), with a *p*-value of 0.0008 (as seen in Figure 3).

### 3.3. Behavioral Studies

#### 3.3.1. OFT

**Figure 4:**
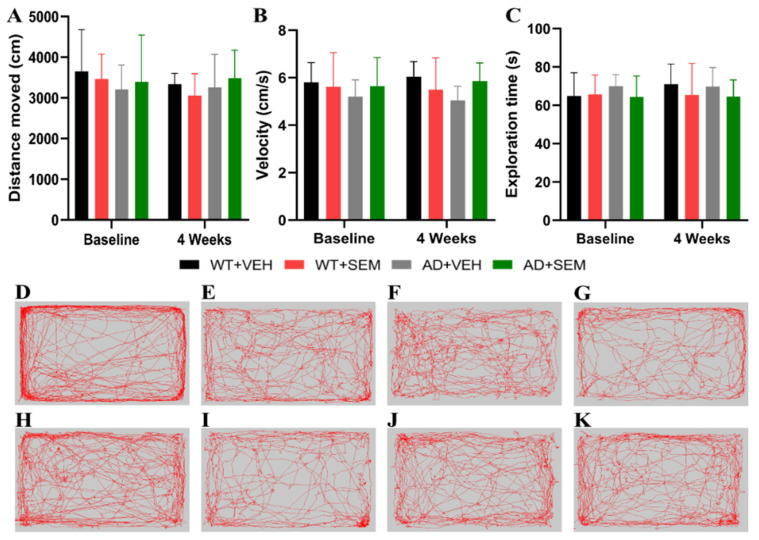
SEM effects on animal motility and exploratory capacity using OFT. (**A**) Distance travelled, (**B**) speed of movement, (**C**) exploration time. (**D**–**K**) Analyzing the recorded arenas and tracking the mice’s path revealed no improvement between baseline: (**D**) WT + VEH, (**E**) WT + SEM, (**F**) AD + VEH, (**G**) AD + SEM; and after 4 weeks of treatment: (**H**) WT + VEH, (**I**) WT + SEM, (**J**) AD + VEH, (**K**) AD + SEM. Data are presented as Mean ± SD.

In terms of the motor and exploratory abilities of the tested animals, the results indicate that SEM administration does not yield significant positive effects. The APP-positive animals treated with SEM exhibited a slight increase in the distance traveled (3484.37 ± 686.97 cm), but this change was not statistically significant when compared to the APP-positive animals receiving only the vehicle (3256.63 ± 813.93 cm), with *p* > 0.05 (Figure 4A).

A similar pattern was observed in the movement speed of the animals; the group of APP-positive animals treated with SEM showed a modest, statistically insignificant increase in speed (5.86 ± 0.76 cm/s) compared to the vehicle-only group (5.04 ± 0.59 cm/s), with *p* > 0.05 (Figure 4B). Additionally, exploratory behavior seemed unaffected by SEM treatment, as both groups spent comparable amounts of time in the center of the arena (Figure 4C).

#### 3.3.2. NORT

The results from the investigation index indicate that prior to SEM administration, animals with AD exhibited a lower investigation index compared with WT animals. Specifically, the WT + VEH group had an index of 76.47 ± 5.98% versus 52.76 ± 7.70% for the AD + VEH group (*p* = 0.0045), and 76.47 ± 5.98% for the WT + VEH group compared to 54.85 ± 10.55% for the AD + SEM group (*p* = 0.0188). Additionally, the WT + SEM group (63.86 ± 5.10%) also showed a significant difference compared to the AD + VEH group (52.76 ± 7.70%), with *p* = 0.0493.

At baseline, no statistically significant differences were observed among the following comparisons: WT + VEH vs. WT + SEM, WT + SEM vs. AD + SEM, and AD + VEH vs. AD + SEM, all with *p* > 0.05.

**Figure 5:**
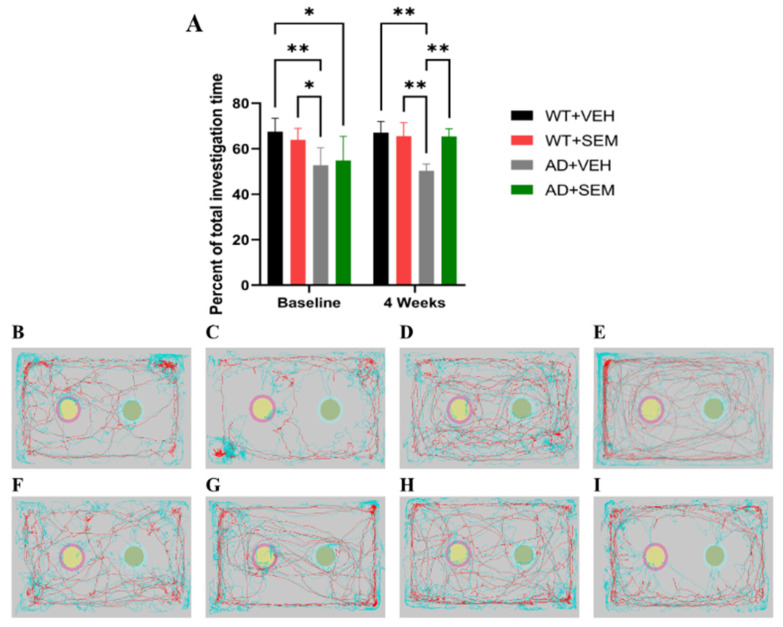
SEM effects on cognition using NORT. (**A**) Percent of total investigation time (%). (**B**–**I**) Analyzing the recorded arenas and tracking the mice’s path revealed statistical differences between the studied groups at baseline: (**B**) WT + VEH, (**C**) WT + SEM, (**D**) AD + VEH, (**E**) AD + SEM; and after 4 weeks of treatment: (**F**) WT + VEH, (**G**) WT + SEM, (**H**) AD + VEH, (**I**) AD + SEM. * *p* < 0.05, ** *p* < 0.01. Data are presented as Mean ± SD.

After 4 weeks of SEM treatment, significant differences emerged between the following groups: WT + VEH (67.08 ± 4.96%) compared to AD + VEH (50.36 ± 2.95%), *p* = 0.0011; WT + SEM (65.54 ± 5.94%) compared to AD + VEH (50.36 ± 2.95%), *p* = 0.0033; and AD + VEH (50.36 ± 2.95%) versus AD + SEM (65.40 ± 3.36%), *p* = 0.0036 (Figure 5). No statistical differences were found when comparing groups at baseline versus 4 weeks after administration.

#### 3.3.3. SCT

**Figure 6:**
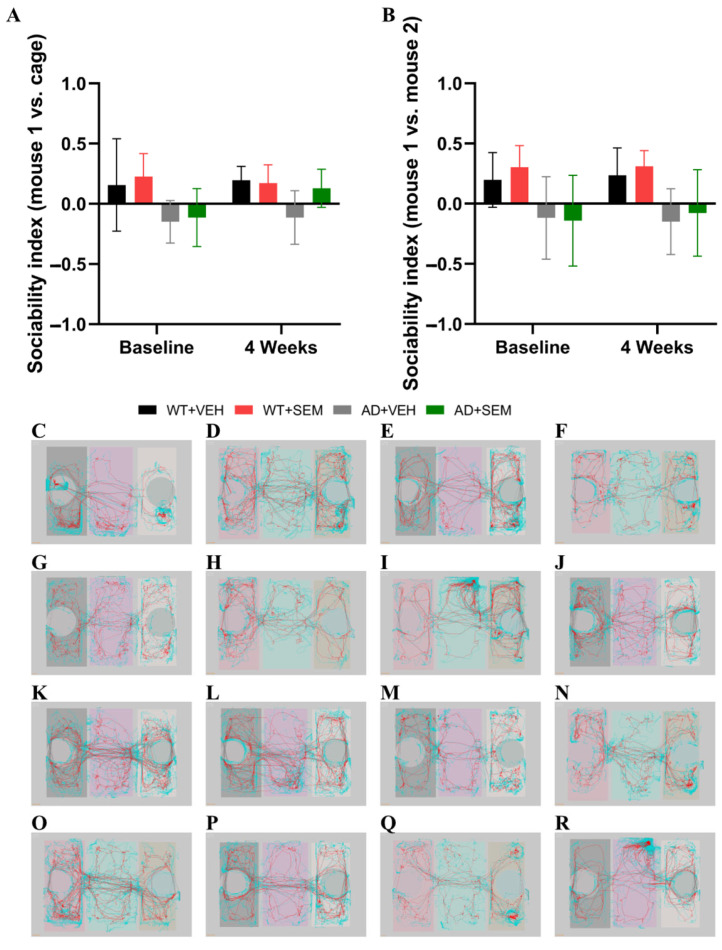
Comparison of the sociability index (**A**) animal vs. object, (**B**) familiar animal vs. new animal using SCT. Analysis of the recorded arenas and tracking the path of the mice revealed no improvement in terms of (**C**–**J**) sociability index (animal vs. object) between baseline, (**C**) WT + VEH, (**D**) WT + SEM, (**E**) AD + VEH, (**F**) AD + SEM, and after 4 weeks of treatment, (**G**) WT + VEH, (**H**) WT + SEM, (**I**) AD + VEH, (**J**) AD + SEM and (**K**–**R**) sociability index (familiar animal vs. new animal) between baseline, (**K**) WT + VEH, (**L**) WT + SEM, (**M**) AD + VEH, (**N**) AD + SEM and after 4 weeks of treatment, (**O**) WT + VEH, (**P**) WT + SEM, (**Q**) AD + VEH, (**R**) AD + SEM. Data are presented as Mean ± SD.

In the analysis of the sociability index versus object at baseline, it was found that animals with AD exhibited a negative index compared to WT animals, although these differences were not statistically significant (*p* > 0.05), as shown in Figure 6A.

Following SEM therapy, the AD animals demonstrated a positive index (0.12 ± 0.15) compared to those treated with the vehicle (−0.11 ± 0.22), as well as to the baseline measurements for the AD + VEH group (−0.14 ± 0.17) and the AD + SEM group (−0.11 ± 0.24). This indicates an improvement in sociability behavior among the AD animals; however, the differences remained statistically insignificant (*p* > 0.05).

Upon calculating the sociability index for the familiar animal versus the newly introduced animal, it was found that animals with AD exhibited a negative index at both baseline and after 4 weeks of SEM therapy. This suggests a strong preference for the newly introduced animal; however, the difference was not statistically significant (Figure 6B).

In contrast, WT animals maintained a positive index throughout the test. Notably, after 4 weeks of SEM therapy, APP-positive mice displayed an index closer to zero (−0.07 ± 0.45) compared to the vehicle-treated group (−0.14 ± 0.27). This may indicate a reduced preference between the two mice, though the differences did not reach statistical significance (*p* > 0.05) (Figure 6B).

#### 3.3.4. 0-MT

**Figure 7:**
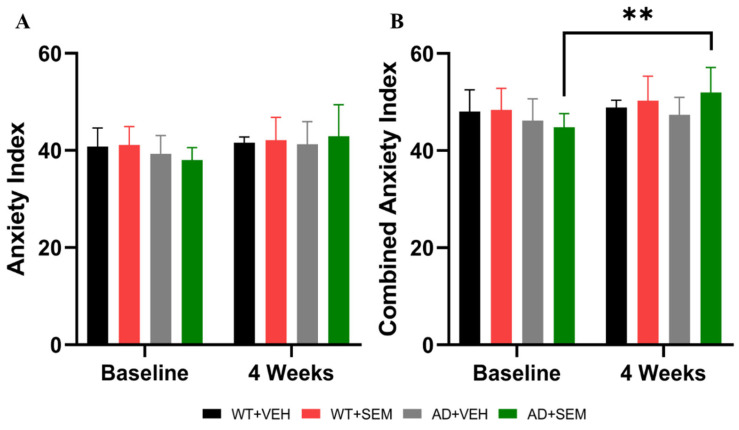
Comparison of the (**A**) anxiety index (calculated based on the time spent by the animal in the open sections of the maze) and (**B**) combined anxiety index (calculated based on the time spent by the animal in the open sections of the maze and the number of transitions between closed sections–open sections) using 0-MT. ** *p* < 0.01. Data are presented as Mean ± SD.

Animal anxiety was assessed by calculating the sociability index based on the duration spent in the open sections of the test (Figure 7A). Additionally, a combined anxiety index was computed, which incorporates both the time spent in the open arms and the number of transitions between open and closed sections (as illustrated in Figure 7B).

The anxiety index did not reveal any statistically significant differences between the animal groups (*p* > 0.05); however, there is a tendency for both APP-positive animals and WT animals treated with SEM to exhibit reduced anxiety-related behavior. The baseline values for the anxiety index were as follows: AD + VEH (39.26 ± 3.82), AD + SEM (38.02 ± 2.53), WT + VEH (40.80 ± 3.84), and WT + SEM (41.14 ± 3.78). After 4 weeks, the values were: AD + VEH (41.27 ± 4.66), AD + SEM (42.93 ± 6.49), WT + VEH (41.55 ± 1.24), and WT + SEM (42.12 ± 4.69).

A similar trend was observed with the combined anxiety index, which did demonstrate statistically significant differences in the AD + SEM group before (44.84 ± 2.78) and after treatment (51.98 ± 5.16), *p* = 0.0013. This indicates that the combined anxiety index, which also accounts for the number of transitions, may provide greater sensitivity in assessing anxious behavior.

## 4. Discussions

The glycemic values observed in this study demonstrate significant differences between the WT groups and the AD transgenic groups both at baseline and after four weeks of treatment. The baseline glycemic values of WT groups were significantly lower compared to AD groups. These results indicate that AD transgenic mice have inherently higher baseline glycemic levels compared to WT mice, suggesting a potential link between amyloid precursor protein (APP) mutations and altered glucose metabolism. After 4 weeks of SEM treatment, the glycemic values in the AD + SEM group were comparable to both WT + VEH and WT + SEM groups. Significant differences remained between AD + VEH and both WT + VEH, and WT + SEM. The AD + SEM group exhibited a statistically significant reduction in blood glucose compared to the AD + VEH group. A marked decrease in glycemic levels was noted within the AD + SEM group from baseline to 4 weeks. These findings suggest that SEM effectively normalizes blood glucose levels in AD transgenic mice, aligning them with those of WT mice. The substantial reduction in glycemia after SEM treatment indicates its potential therapeutic benefit in managing glucose metabolism in AD pathology.

According to the obtained results, our study also confirms the data found in the specialized literature [22,35], more specifically the fact that APP positive animals present higher blood glucose levels than WT animals.

Before the initiation of SEM injection, no statistical differences were observed between the four groups of studied animals. However, APP-positive animals tended to have lower weights compared to WT animals, which has also been shown by other researchers [22,36,37,38]. The weight loss effect of SEM is well documented in the literature in rodents [39,40,41].

The WT + VEH group showed a significant weight gain from baseline to 4 weeks. Conversely, weight loss was observed in the WT + SEM group and AD + SEM group from baseline to 4 weeks. After 4 weeks, the AD + SEM group had significantly lower weights compared to both the AD + VEH group, *p* = 0.0348, and the WT + VEH group. The weight gain in the WT + VEH group suggests that normal physiological conditions (without any pharmacological intervention) lead to natural growth and weight gain. In contrast, the weight loss observed in the WT + SEM and AD + SEM groups indicates that SEM might influence metabolic processes leading to weight reduction. This is consistent with SEM’s known effects in humans, where it is used to aid weight loss in addition to controlling blood glucose levels.

The findings indicate that SEM serves a dual purpose in AD transgenic mice by enhancing glycemic control and promoting weight loss. The normalization of blood glucose levels in AD mice following SEM treatment highlights its potential therapeutic value in addressing metabolic dysfunction linked to AD. While the weight loss resulting from SEM administration can be advantageous for combating obesity, it is important to approach this aspect with caution in the context of AD, as maintaining an adequate body weight is vital for overall health and functionality.

Various behavior tests have been used to assess SEM’s administration impact in murine models of neurodegenerative disorders like AD (OFT, NORT, Y-Maze Test) [22] and Parkinson’s disease (OFT, Rotarod Test, Vertical Pole Test, Footprint Gait Test) [42,43,44].

In a review paper from 2024, it was described that in preclinical models of AD, SEM administration has been associated with alleviated neuroinflammation and decreased amyloid-β plaque deposition. Also, SEM-treated animals exhibited ameliorated behavioral deficits related to anxiety and social interaction, and improvements in memory retention tasks and spatial learning [45].

The administration of SEM did not influence the exploratory capacity of the animals, the obtained data being consistent with those described in the study published by Wang et al. [22].

The OFT results indicate that SEM administration did not significantly enhance the motor and exploratory abilities of AD transgenic mice. Positive APP animals treated with SEM showed a slight increase in distance traveled compared to those receiving the vehicle, but this difference was not statistically significant. A modest increase in movement speed was observed in the SEM-treated group compared to the vehicle group, yet this was also statistically insignificant. Both groups (SEM-treated and vehicle-treated) spent similar amounts of time in the center of the arena, indicating no significant improvement in exploratory behavior due to SEM administration. The lack of significant improvement in distance traveled and movement speed suggests that SEM does not notably enhance motor functions in AD transgenic mice. This aligns with the known primary effects of SEM, which are more related to metabolic regulation rather than direct neurological or motor enhancements. The consistency in exploratory behavior, as shown by similar time spent in the center of the arena, suggests that SEM does not affect anxiety-like behaviors or curiosity-driven exploration in AD mice. The center time in the OFT is often used as an indicator of anxiety, where less anxious animals typically spend more time exploring the center. Since no differences were observed, it can be inferred that SEM does not influence anxiety levels in these animals.

While SEM shows significant metabolic benefits, its impact on behavior, particularly motor and exploratory activity, appears limited. This might indicate that while SEM effectively manages systemic metabolic dysfunctions in AD, it does not address the neurobehavioral deficits associated with AD pathology, at least in the aspects of motor activity and exploration measured by the OFT.

These findings underscore the importance of a multifaceted therapeutic approach for AD, where metabolic regulation (e.g., through SEM) might need to be combined with treatments targeting cognitive and motor functions directly. Considering SEM’s efficacy in managing glycemic levels, it might be beneficial as part of a combination therapy approach for AD, where metabolic stability is a foundational treatment, supplemented by other interventions aimed at improving cognitive and behavioral outcomes.

Our results of the NORT confirm previous research showing that SEM administration has beneficial effects on the short-term memory of APP-positive animals, which are able to recognize the novel object, compared to APP-positive animals that received only the vehicle [22].

At baseline, the AD transgenic mice had a significantly lower investigation index compared to the WT mice. This indicates impaired cognitive function and memory recognition in AD mice, as they spent less time investigating the novel object compared to the familiar one. The lack of significant differences within the same groups (WT or AD) before SEM administration suggests that the baseline cognitive functions were comparable among the respective groups, ensuring a controlled and unbiased comparison for post-treatment analysis. After 4 weeks of SEM therapy, the investigation index of AD mice treated with SEM showed a significant improvement compared to those treated with vehicle. This suggests that SEM has a positive effect on cognitive function in AD transgenic mice, enhancing their ability to recognize and investigate a novel object.

No statistical differences were observed within each group from baseline to 4 weeks post-treatment. This indicates that while SEM improved cognitive function in AD mice relative to the vehicle-treated group, the individual changes within each group from baseline to post-treatment did not reach statistical significance. This could be due to variability within groups or the duration of the study not being long enough to detect significant within-group changes. The lower baseline investigation index in AD mice confirms cognitive impairment associated with AD pathology. This impairment affects their ability to discriminate between familiar and novel objects, which is a hallmark of memory deficits in AD.

SEM appears to enhance cognitive function in AD mice, as evidenced by the significant improvement in the investigation index after 4 weeks of treatment. The treated AD mice showed similar cognitive performance to WT mice, indicating a potential therapeutic effect of SEM on cognitive deficits in AD. While the study does not explore the mechanisms, the observed cognitive benefits could be due to improved metabolic regulation, reduced neuroinflammation, or direct neuroprotective effects of SEM. These findings support the potential of SEM as a therapeutic agent for cognitive impairment in AD. The normalization of cognitive function in AD mice suggests that SEM could mitigate some of the cognitive symptoms associated with AD, potentially through mechanisms beyond glucose regulation.

In SCT, AD mice had a negative sociability index compared to WT mice, indicating lower sociability, but this difference was not statistically significant. This suggests that AD mice, even at baseline, exhibit reduced social interactions compared to WT mice, which is a common characteristic of AD pathology affecting social behavior. After SEM therapy, AD mice showed a positive sociability index compared to AD mice treated with the vehicle and baseline AD mice. However, these differences were not statistically significant. This positive shift in the sociability index after SEM therapy indicates an improvement in social behavior among AD mice, suggesting that SEM may enhance sociability, although the changes were not statistically significant. AD mice showed a negative sociability index both at baseline and after 4 weeks of SEM therapy, indicating a strong preference for the newly introduced animal over the familiar one. This preference remained without statistical significance.

WT mice consistently showed a positive index, indicating a balanced sociability towards both familiar and newly introduced animals. After 4 weeks of SEM therapy, APP-positive mice had an index closer to 0 compared to vehicle-treated animals, suggesting a lack of preference between the two mice, but again, these differences were not statistically significant.

Regarding 0-MT, the negative baseline sociability index in AD mice confirms the reduced sociability commonly seen in AD pathology. This reduction in social interaction could be linked to cognitive impairments and changes in brain regions associated with social behavior. The shift towards a positive sociability index after SEM therapy, despite not being statistically significant, suggests a trend towards improved social behavior in AD mice. SEM may help alleviate some of the social deficits associated with AD, potentially through its metabolic effects or direct action on brain function. The strong preference for newly introduced animals in AD mice, as indicated by a negative sociability index, may reflect a disruption in social memory or a heightened novelty-seeking behavior, which is not significantly altered by SEM treatment. The WT mice’s positive index throughout the test indicates a stable social memory and balanced sociability, contrasting with the altered social preference in AD mice. The movement towards a neutral sociability index after SEM therapy suggests a possible normalization of social preferences in AD mice, even if not statistically significant. This trend is worth noting as it may indicate an underlying improvement in social cognition.

The baseline anxiety index showed no significant differences between the groups in 0-MT. This suggests that at baseline, both AD transgenic and WT mice had comparable levels of anxiety-like behavior, indicating a consistent starting point for evaluating the effects of SEM therapy. There were no statistically significant differences observed in the anxiety index post-treatment. However, a trend towards decreased anxiety-like behavior was noted in both APP-positive and SEM-treated groups, suggesting a potential anxiolytic effect of SEM, albeit not statistically significant.

The significant change observed in the combined anxiety index for the AD + SEM group underscores the importance of using comprehensive measures to assess anxiety. The combined index’s sensitivity to both the time spent in open sections and transitions between sections offers a more accurate reflection of anxiety-like behaviors and the impact of therapeutic interventions.

The trends observed in both the standard and combined anxiety indices suggest that SEM may help alleviate anxiety-like behaviors in AD mice. While not statistically significant in all measures, the observed trends are promising and warrant further investigation. The significant findings in the combined anxiety index highlight the value of using multifaceted behavioral assessments. This approach can provide a more detailed understanding of the effects of treatments like SEM on complex behaviors such as anxiety.

Although promising, our study has its limitations, and more extensive studies are needed to confirm the observed effects. While our findings suggest potential directions for future treatment strategies, this research is a pilot study. Longer treatment durations, larger sample sizes, or combined therapies should yield more conclusive results.

## 5. Conclusions

The findings from this study suggest that SEM may provide a therapeutic approach to alleviate metabolic disturbances associated with AD. Future research should investigate the long-term effects of SEM on cognitive function and overall health in AD models, while considering the trade-off between its glycemic benefits and effects on body weight. The SEM treatment notably reduced weight in AD transgenic mice compared to the control group, aligning with its established role in promoting weight loss and improving glucose control.

However, SEM did not significantly improve motor and exploratory abilities in AD transgenic mice, as demonstrated by the OFT. Nevertheless, SEM administration led to a significant increase in the investigation index compared to the vehicle group, suggesting potential cognitive benefits as observed in the NORT. Although SEM therapy resulted in a positive, yet statistically non-significant, change in the sociability index, it points to possible improvements in social behavior as measured in the SCT. Trends post-treatment also suggested a potential anxiolytic effect of SEM, particularly reflected in the combined anxiety index for the AD mice during the 0-MT.

These results underscore the need for multifaceted therapeutic strategies for AD, integrating metabolic management with interventions aimed at cognitive and behavioral impairments. This study is a first step toward understanding the potential for such combined interventions in AD, with future research necessary to confirm and extend these early findings.

## Data Availability

Data are contained within the article.
